# Quantifying State-Dependent Control Properties of Brain Dynamics from Perturbation Responses

**DOI:** 10.1523/JNEUROSCI.0364-25.2025

**Published:** 2025-12-19

**Authors:** Yumi Shikauchi, Mitsuaki Takemi, Leo Tomasevic, Jun Kitazono, Hartwig R. Siebner, Masafumi Oizumi

**Affiliations:** ^1^Graduate School of Arts and Sciences, The University of Tokyo, Tokyo 153-8902, Japan; ^2^Medical Institute of Developmental Disabilities Research, Showa Medical University, Tokyo 157-8577, Japan; ^3^Graduate School of Advanced Science and Engineering, Hiroshima University, Hiroshima 739-8527, Japan; ^4^Danish Research Centre for Magnetic Resonance, Centre for Functional and Diagnostic Imaging and Research, Copenhagen University Hospital Amager and Hvidovre, Hvidovre 2650, Denmark; ^5^Department of Psychiatry and Psychotherapy, University of Regensburg, Regensburg 93053, Germany; ^6^Department of Human Sciences, Institute of Psychology, University of the Bundeswehr Munich, Neubiberg 85579, Germany; ^7^Graduate School of Data Science, Yokohama City University, Kanagawa 236-0027, Japan; ^8^Department of Neurology, Copenhagen University Hospital Bispebjerg and Frederiksberg, Copenhagen NV 2400, Denmark; ^9^Department of Clinical Medicine, Faculty of Health and Medical Sciences, University of Copenhagen, Copenhagen N 2200, Denmark

**Keywords:** concurrent TMS-EEG measurement, data-driven control, motor execution, motor imagery, network controllability, resting state

## Abstract

The brain can be conceptualized as a control system facilitating transitions between states, such as from rest to motor activity. Applying network control theory to measurements of brain signals enables characterization of brain dynamics through control properties. However, most prior studies that have applied network control theory have evaluated brain dynamics under unperturbed conditions, neglecting the critical role of external perturbations in accurate system identification. In this study, we combine a perturbation input paradigm with a network control theory framework and propose a novel method for estimating the controllability Gramian matrix in a simple, theoretically grounded manner. This method provides insights into brain dynamics, including overall controllability (quantified by the Gramian’s eigenvalues) and specific controllable directions (represented by its eigenvectors). As a proof of concept, we applied our method to transcranial magnetic stimulation-induced electroencephalographic responses across four motor-related states and two resting states. We found that states such as open-eye rest, closed-eye rest, and motor-related states were more effectively differentiated using controllable directions than overall controllability. However, certain states, like motor execution and motor imagery, remained indistinguishable using these measures. These findings indicate that some brain states differ in their intrinsic control properties as dynamical systems, while others share similarities. This study underscores the value of control theory-based analyses in quantitatively how intrinsic brain states shape the brain’s responses to stimulation, providing deeper insights into the dynamic properties of these states. This methodology holds promise for diverse applications, including characterizing individual response variability and identifying conditions for optimal stimulation efficacy.

## Significance Statement

The brain can be viewed as a control system transitioning between states, such as from rest to motor activity. Previous studies using network control theory mostly evaluated brain dynamics without external perturbations, neglecting their role in accurate system identification. This study integrates perturbation inputs with network control theory to propose a method for estimating the controllability Gramian, thereby providing insights into brain dynamics. We applied this approach to transcranial magnetic stimulation-induced electroencephalographic responses in motor-related and resting states. Our findings show that controllable directions (eigenvectors) allow better discrimination between states than overall controllability. Our method can quantitatively assess brain state differences, and has potential applications in characterizing individual response variability and optimizing stimulation efficacy.

## Introduction

Network control theory has been applied to neuronal networks to deepen our understanding of complex cognitive processes in neuroscience (Gu et al., 2015; Medaglia et al., 2017). The approach models the brain as a control system, highlighting its ability to transition between states, such as from motor activity to rest. It introduces mathematically-grounded and interpretable measures to analyze brain activity as multidimensional time series across different regions. The core concept is controllability, which refers to the brain’s capacity to shift its dynamics from one state to another via internal or external inputs. Mathematically, controllability is defined using the controllability Gramian, a matrix that captures how the brain’s state changes under the influence of these inputs over time. This framework offers a more holistic understanding of neural processes compared with fragmented approaches focusing on specific activity features.

Pioneering studies have used network control theory to explore how the brain’s structural connectivity constrains its dynamics. They have provided mechanistic explanations for transitions between cognitive states, identifying key areas of the brain that can facilitate these transitions (Gu et al., 2015) and providing insight into optimal trajectories between states, such as moving from high activity in the default mode network to high activity in sensorimotor systems (Gu et al., 2017). These studies have significantly advanced our understanding of how the structural connectivity of the brain governs its control capabilities.

Our study aims to advance the understanding of brain dynamics by shifting the focus from structural connectivity to dynamic neural activity data and examination of state-dependent changes in control properties. Previous research has largely relied on passive data (i.e., data without external inputs; Deng et al., 2022; Kawakita et al., 2022; Kamiya et al., 2023), limiting its ability to fully characterize the brain’s dynamic nature. To address this limitation, it is considered essential to apply perturbative inputs is essential for accurate system identification (Jakowluk, 2024; Ogino et al., 2025), enabling a more precise characterization of brain dynamics.

Although not explicitly grounded in control theory, the importance of perturbative inputs has been demonstrated in studies investigating cognitive states such as working memory (Rose et al., 2016), states of consciousness (Casali et al., 2010, 2013; Lee et al., 2022), and spatial attention (Okazaki et al., 2020). However, while these studies underscore the value of perturbative approaches, their reliance on one-dimensional, global measures like the perturbational complexity index (Casali et al., 2010, 2013; Lee et al., 2022) limits the granularity of insights because these measures primarily capture the overall complexity or the degree of signal propagation in brain dynamics.

In this study, we integrate perturbative inputs with network control theory to develop a simple, theoretically grounded method for quantifying multivariate, state-dependent changes in control properties. The method estimates the controllability Gramian directly from neural time series following impulse-like inputs, without estimating the connectivity matrix *A* or the input matrix *B* (Eq. 1). In this representation, the Gramian’s eigenvalues index overall controllability (magnitude), and its eigenvectors identify the directions along which dynamics can be effectively influenced. As a proof of concept, we applied the method to single-trial, single-pulse transcranial magnetic stimulation (TMS)-electroencephalographic (EEG) data spanning four motor-related task conditions and two resting conditions (Table 1). Treating brief (<2 ms) TMS pulses as impulses justifies this estimation and keeps the analysis focused on short-latency components that minimize peripheral effects (Biabani et al., 2019; Conde et al., 2019; Rocchi et al., 2021). The high temporal resolution of EEG supports robust characterization differences between motor-related and resting conditions in both the magnitude and the directions of controllability.

**Table 1.** Experimental conditions

Condition	Abbreviation	Condition overview (TMS timing)
Motor execution	ME	Right index finger isometric abduction for 3 s
		(1.5–2.5 s after the task onset)
Motor imagery	MI	Imagery of the motor execution task
		(1.5–2.5 s after the task onset)
After motor execution	AfterME	After the motor execution task
		(4–5 s after the task onset)
Resting state with open eyes	OE	(Every 4–6 s)
Resting state with closed eyes	CE	(Every 4–6 s)
Ipsilateral motor execution	iME	Left index finger isometric abduction for 3 s
		(1.5–2.5 s after the task onset)

## Materials and Methods

### Theoretical background to control theory

In this study, we consider a linear auto-regressive model as a model of multidimensional brain dynamics and quantify the control properties of the system given some external input. Specifically, we consider the following model:

x(t)=Ax(t−1)+Bu(t−1),(1)

where $x(t)\in R^{n}$ is a *n*-dimensional vector of the brain activity at time *t*,

$$x(t)=[x_{1}(t),x_{2}(t),\ldots ,x_{n}(t){]}^{T},\;(2)$$

**Figure 1.**
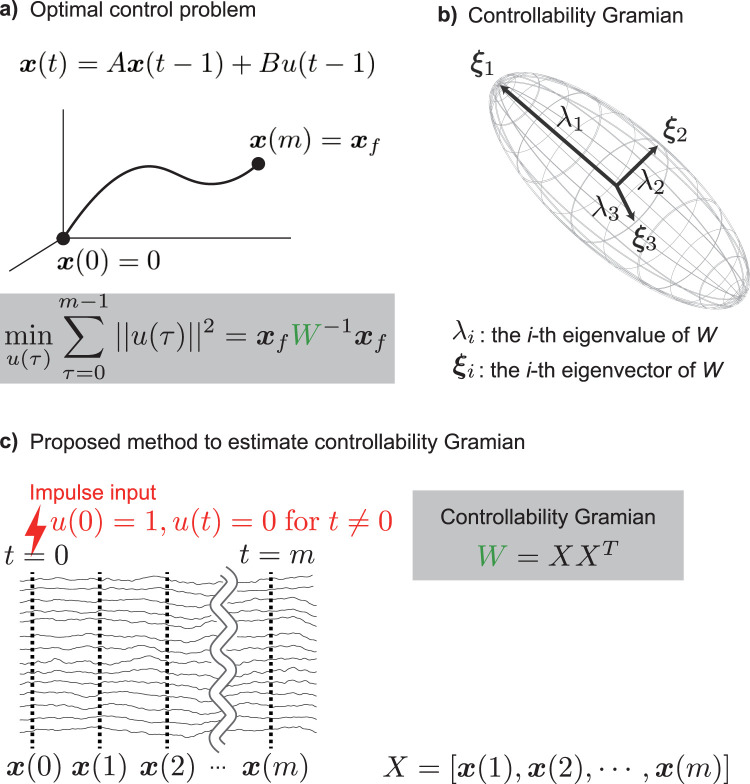
Schematic illustration of the controllability Gramian. ***a***, In a first-order auto-regressive model, the transition from the initial state ***x***(0) to the final target state ***x***(*m*) at minimum cost is realized if the inverse of the controllability Gramian is known, as the equation in the gray shaded box shows. ***b***, The Gramian *W* is an *n*-dimensional hyperellipse, encompassing all spatial patterns that appear in the controllability matrix. *n* is the measurement dimension as long as the measurement dimension does not exceed the number of time points. The major axis shows the first eigenvector ***ξ***_1_. The second and third eigenvectors ***ξ***_2_ and ***ξ***_3_ are shown as arrows orthogonal to the first eigenvector. The length of each axis corresponds to the magnitude of change as an eigenvalue *λ*_·_, and the direction of a specific spatial pattern as an eigenvector ***ξ***_·_. ***c***, Assuming that the control input is an impulse, the controllability matrix can be regarded as the measurement signals *X*, as the equation in the gray shaded box shows. Under this assumption, we obtain the controllability Gramian without estimating the connectivity matrix *A* and input matrix *B*. Each line on the left indicates the time series measured by each sensor.

*A* is the *n* × *n* connectivity matrix that describes the interaction between the elements, u(t−1)∈R is a one-dimensional external input at time *t* − 1, and *B* is an *n* × 1 input matrix describing how the control input *u* affects the brain activity *x*. We consider the dynamics from the time *t* = 0 to *t* = *m* (Fig. 1).

We wish to characterize both to what extent and how the external input *u* can control the system. To this end, the most important quantity to quantify is the so-called controllability Gramian. We will briefly explain the definition and meaning of the controllability Gramian, but for further details please refer to Brunton and Kutz (2019).

The controllability Gramian *W* is defined using the controllability matrix C as follows:

$$W={CC}^{T},\;(3)$$

where:

$$C=[B,AB,A^{2}B,\ldots ,A^{m-1}B].\;(4)$$

The controllability Gramian is important because it gives the solution to an example optimal control problem is: given the initial state ***x***(0) = ***x***_*s*_ and the final target state ***x***(*m*) = ***x***_*f*_, find the most efficient control inputs in terms of minimum cost.

Here, to simplify the expression, we introduce a new variable $\tilde{x}(t)$, which is the time series ***x***(*t*) subtracted from ***x***(0), $\tilde{x}(t)\equiv x(t)-x(0)$, so that $\tilde{x}(0)=0$ (Fig. 1*a*). Under this notation, the optimal control problem is formulated by:

$$\min_{u(\tau )}\left\{\sum_{\tau =0}^{m-1}\|u(\tau ){\|}^{2}\;|\;\tilde{x}(0)=0,\tilde{x}(m)={\tilde{x}}_{f}\right\},\;(5)$$

where ${\tilde{x}}_{f}=x_{f}-x(0)$. By solving this constrained optimization, we can find the minimal cost and the optimal control input according to:

$$\min_{u(\tau )}\sum_{\tau =0}^{m-1}\|u(\tau ){\|}^{2}={\tilde{x}}_{f}W^{-1}{\tilde{x}}_{f},\;(6)$$

$$u(\tau )=B^{T}(A^{T}{)}^{m-1-\tau }W^{-1}{\tilde{x}}_{f}.\;(7)$$

The equation for the minimum control cost (Eq. 6) gives the exact mathematical meaning of the controllability Gramian *W* in terms of the optimal control framework. Assuming that the controllability Gramian has a set of eigenvalues *λ*_*i*_ and corresponding normalized eigenvectors ***ξ***_*i*_, then, from Equation 6, we can easily see that the larger the eigenvalue is, the larger we can move the state of the system in the corresponding direction of the eigenvector. More precisely, given a fixed amount for the sum of the squared control input, i.e., $\sum_{\tau =0}^{m-1}\|u(\tau ){\|}^{2}=1$, the squared distance we can move in the direction of an eigenvector is exactly given by its eigenvalue. This can be shown by setting the final target state to ${\tilde{x}}_{f}=r_{i}\xi_{i}$, where *r*_*i*_ corresponds to the distance to move, and substituting this final state into Equation 6 gives $r_{i}^{2}=\lambda_{i}$.

In summary, by ordering the eigenvectors of the controllability Gramian according to the highest to lowest eigenvalues from highest to lowest, we can tell which direction we can easily move with the optimal control input: the direction of the largest eigenvalue corresponds to the most easily controllable direction, that of the second largest eigenvalue corresponds to the second most easily controllable direction, and so on (Fig. 1*b*). Note that the degree of ease of control is defined by the hypothetical optimal control input, not by the actual impulse input. In Figure 1*b*, we illustrate this visually by considering the controllability Gramian as a hyperellipse, whose longest axis corresponds to the first eigenvector, whose second longest axis corresponds to the second eigenvector, and so on. In this study, we characterize the control property of the brain dynamics in this way.

### Experimental design and statistical tests

#### Participants

Seventeen healthy volunteers (11 women, age range: 18–29 years) participated in the experiment. All participants had slept between 6.5 and 11.0 h the night before the experiment, were right-handed as assessed by the Edinburgh handedness inventory (Oldfield, 1971; range: 65–100), did not smoke regularly except for one participant, had no history of neurological or psychiatric diseases, and had no contraindications to TMS. After receiving written and oral information about the experiment, the participants gave written informed consent before testing. All TMS-EEG data were collected between December 2016 and March 2017 as a part of a TMS-EEG project approved by the local ethics committee of the capital region of Denmark (H-15008824). The study was carried out under the guidelines of the Helsinki Declaration.

#### EEG and EMG recording

EEG and electromyographic (EMG) signals were recorded with a NeuroOne Tesla system (Mega Electronics) with a 63-channel equidistant M10 EEG cap (EASYCAP) and surface EMG electrodes (Ambu Neuroline 710). EMG electrodes were placed on the first dorsal interosseous muscle on the right-hand, using a bipolar belly-tendon montage and a ground electrode mounted at the right ulnar styloid process. All EEG electrodes, including a ground electrode and a reference electrode of the cap, were prepared using NuPrep Skin Prep gel and ABRALYT HiCl EEG electrode gel (EASYCAP). EEG electrode impedance was maintained below 5 kΩ throughout the recording periods. EEG and EMG signals were digitized at 5 kHz with a 1,250-Hz low-pass filter and DC filter.

#### Neuro-navigated TMS-EEG

TMS was applied over the hand area of the primary motor cortex (M1-HAND) of the left hemisphere using a MagPro X100 with an MC-B70 figure-of-eight coil (MagVenture). The coil was placed tangentially on the scalp, with the handle directed posteriorly at a 45° angle to the sagittal midline. To reduce TMS artifacts in the EEG signals, a soft silicone rubber sheet (1.5-mm thick) was placed between the coil and the EEG electrodes. Biphasic pulses, in which the second phase induced a posterior-to-anterior current in the cortex, were employed. Continuous monitoring of the TMS coil placement was ensured via a frameless stereotaxic neuro-navigation system (Localite). The root mean square of the disparity from the co-registered landmark over the M1-HAND was kept under 2 mm throughout the experiment.

The individual M1-HAND hotspot was identified as the coil position eliciting the most consistent and prominent motor-evoked potentials (MEPs) using suprathreshold stimulation intensity while the subject was at rest. This hotspot was marked in the neuro-navigation system. The resting motor threshold was determined after mounting the EEG cap using maximum-likelihood parameter estimation with the sequential testing approach while participants remained at rest (Awiszus, 2003). MEPs exceeding 50 μV were classified as “responding.”

#### TMS-EEG measurements

Participants underwent six conditions (Table 1), during which a single-pulse of TMS was applied to M1-HAND at a stimulation intensity of 120% of the resting motor threshold for each trial. One hundred trials were carried out per condition. Participants sat in a comfortable armchair, and auditory noise masking was consistently applied throughout the TMS-EEG measurements using in-ear headphones (Insert Earphone 3A 410–3002, 3M systems). Consistent with previous procedures, we employed specific time- and frequency-varying sounds for noise masking (Herring et al., 2015; Conde et al., 2019; Beck et al., 2024). The sound pressure for noise masking was individually adjusted to a level where participants could not hear the click sound of the TMS pulse with the TMS coil placed on their M1-HAND or until they reached their upper threshold for comfort.

Measurements commenced with either the OE or CE conditions described in Table 1, with the order of these conditions being pseudo-randomized across participants. Participants placed their right-hand, palm side down, on the armrest and remained at rest. In the OE condition, participants were instructed to keep their eyes-open and fixate on a fixation cross, while the TMS pulse was administered every 4–6 s. In the CE condition, participants were instructed to keep their eyes-closed, and the TMS pulse was delivered every 4–6 s.

Following the OE and CE conditions, three conditions involving right-hand movement or motor imagery (ME, MI, and AfterME) were conducted. In these three conditions, each trial began with the presentation of open gray and filled blue circles (ME and AfterME) or an open blue circle (MI) on a computer monitor placed in front of the participants. After the presentation of the two circles, participants performed 3 s of isometric abduction of the right index finger at 10% of maximum voluntary contraction. The size of the filled blue circle increased depending on the force level applied. Participants were instructed to match the size of the two circles as quickly as possible, meaning that the applied force level corresponded to 10% of the maximum voluntary contraction. In the MI condition, after presentation of the open blue circle, participants performed 3 s of kinesthetic motor imagery of the ME content without moving their right-hand. After 3 s, a gray cross appeared on the monitor, which prompted participants to cease motor tasks and return to a resting state until the next trial began. TMS was administered 1.5–2.5 s from the start of each trial in the ME and MI conditions and 4–5 s from the start in the AfterME condition. The ME, MI, and AfterME conditions were pseudo-randomized per trial and were paused multiple times to allow participants a short break and prevent mental fatigue.

Finally, we conducted the iME condition, in which participants were asked to perform the ME content with their left-hand. TMS was administered 1.5–2.5 s from the start of each trial.

#### EEG data preprocessing

Offline data analysis was performed using with the FieldTrip toolbox for EEG and MEG analysis (Oostenveld et al., 2011). Our proposed method compresses the time series data in the temporal dimension (see “Theoretical background to control theory”). Since high-amplitude components can have lasting effects beyond their occurrence, the following preprocessing steps were implemented to minimize the impact of cranial muscle artifacts as much as possible. Initially, TMS-induced artifacts were removed from EEG signals using the TESA methods (Rogasch et al., 2017) with MATLAB software (MathWorks). In brief, the TMS-EEG data underwent the following processing steps: (1) removal of noisy channels; (2) epoching of data from −1 to +1 s relative to the TMS pulse for the OE and CE conditions and from −1 to +6 s relative to the onset of the motor task for the other conditions; (3) baseline correction with data from −500 to −110 ms relative to the TMS pulse for the OE and CE conditions or relative to the motor task onset for other conditions; (4) removal of periods containing TMS pulse artifacts and TMS-evoked muscle activity (−2 to 15 ms relative to the TMS pulse); (5) interpolation of missing data around the TMS pulse with constant amplitude values; (6) removal of noisy trials; (7) independent component analysis (ICA) and removal of components representing muscle artifacts; (8) replacement of data of the removed window (−2 to 15 ms relative to the TMS pulse) by interpolation using a cubic function; (9) second-order Butterworth filtering (1–80 Hz); (10) notch filtering; (11) replacement of data from the removed window with constant amplitude values; (12) ICA to remove residual artifacts; (13) spatial interpolation of the rejected channels; (14) common average re-referencing; and (15) linear interpolation of the 0–20 ms signal was applied to completely eliminate the residual effects of artifacts. As a result of the above preprocessing, an average of 2.25 (SD 1.51, range 0–8) channels, an average of 13.99% (SD 12.10, range 0 − 58) of trials per session were excluded. Through these preprocessing steps, we obtained TMS-induced artifact-free EEG data.

In this study, the preprocessing steps used in TMS-EEG analysis could have influenced the results, particularly in terms of distinguishing between conditions. One such preprocessing method is ICA, which can affect the analysis by removing certain components. In the sensitivity analysis, we systematically varied the threshold used to reject ICA components based on the peak amplitude (*Z*-score) of EEG signals immediately following TMS (Shikauchi et al., 2025; see Supplementary Text, “Robustness check using an independent dataset” and Fig. S1). Specifically, we applied rejection thresholds of *Z* = 1, 3, 6, and 15, as well as a setting in which ICA component removal was not performed (“None”). For each threshold setting, we computed the controllability Gramian from the resulting data, and extracted its eigenvalues and eigenvectors. We then calculated trial-to-trial distance matrices for each of these quantities and summarized the overall variability by computing the mean distance across trials. This allowed us to assess how sensitive the estimated controllability properties are to ICA thresholding parameters in a reproducible analysis pipeline.

#### Extracting the controllability matrix from single-shot TMS-EEG

We then extracted the controllability matrix corresponding to Equation 13 from the TMS-induced artifact-free data. Focusing on cortical responses, signals with short latencies up to 60 ms were included in the analysis, thereby avoiding peripheral responses, which have a strong influence on long latencies (Biabani et al., 2019; Rocchi et al., 2021). The analysis was performed according to the following steps: (1) scaling by the standard deviation of signals from −200 to −5 ms before stimulation to correct for differences in signal values per electrode; (2) downsampling to 1,070 Hz to align the number of time points with the number of channels; (3) baseline correction with the values immediately previous to satisfy the assumption that $\tilde{x}(0)=0$; and (4) segmentation from 1 to 60 ms from TMS onset. This resulted in a 63-channel × 63-time points data matrix of EEG signals for each trial. There is no need to estimate the connectivity matrix *A* and the input matrix *B* in Equaion 1.

When the analysis was performed over longer time windows, i.e., 1–120 and 1–300 ms, the downsampling frequency was set to 530 and 220 Hz, respectively, so that the controllability matrix was a square matrix.

#### Definition of pairwise distance

We treated the single-shot TMS-EEG data as the controllability matrix described in the previous section, from which we computed the controllability Gramians (Eq. 3). For each trial, the resulting controllability Gramian was further analyzed through its eigenvalues and eigenvectors. To characterize trial-by-trial variations, we quantified pairwise distances between trials on the basis of these properties. This analysis was implemented in MATLAB using in-house code and the publicly available SPDtoolbox on GitHub (https://github.com/kyoustat/papers; You and Park, 2021).

We used the following three distance measures.

##### Gramian-based distance

The controllability Gramians are considered to be on the symmetric positive definite (SPD) space, which is a Riemannian manifold. For any two SPD matrices *w* and $w^{\prime }$, the affine-invariant Riemannian metric between them is defined as Bhatia (2009):

$$D^{\mathrm{total}}(w,w^{\prime })=||\mathrm{Log}(w^{-1}w^{\prime })|{|}_{F},\;(8)$$

where Log(*a*) is the matrix logarithm of a square matrix *a*, and ||*a*||_*F*_ is the Frobenius norm of *a*.

When focusing only on up to the *m*th eigenmode (i.e., excluding the eigenmodes after the *m* + 1st eigenmode), the reconstructed controllability Gramian $\hat{w}$ could be obtained according to the following. The original controllability Gramian *W* was decomposed into its eigenvalues and eigenvectors according to:

$$W=U\Lambda U^{T},$$

where *U* is the matrix of eigenvectors and Λ is the diagonal matrix containing the eigenvalues *λ*_1_, *λ*_2_, …, *λ*_*n*_ in descending order. To achieve dimensionality reduction, we reconstructed the Gramian matrix using only the top *m* eigenvalues *λ*_1_, *λ*_2_, …, *λ*_*m*_ and their corresponding eigenvectors. The reconstructed Gramian $\hat{W}$ is given by:

$$\hat{W}=U_{m}\Lambda_{m}U_{m}^{T},$$

where *U*_*m*_ consists of the first *m* columns of *U*, and Λ_*m*_ is the diagonal matrix containing the first *m* eigenvalues. This allowed us to approximate the original Gramian in a lower-dimensional space.

##### Eigenvalue-based distance

Eigenvalues are considered as points on the complex number plane and Euclidean distance is used. The inter-trial distance of the average controllability was calculated using the following equation:

$$D^{\mathrm{magnitude}}=\frac{\left|\sum_{i=1}^{m}\lambda_{i}-\sum_{i=1}^{m}\lambda_{i}^{\prime }\right|}{\sum_{i=1}^{m}\lambda_{i}+\sum_{i=1}^{m}\lambda_{i}^{\prime }},$$

where *λ*_*i*_ is the *i*-th eigenvalue of one trial, and $\lambda_{i}^{\prime }$ corresponds to that of another trial. *m* represents the number of eigenmodes, which is typically equal to the number of channels (*m* = *n* = 63) when all dimensions are utilized. However, *m* can be set to a smaller value than *n* when focusing only on the upper eigenmodes.

##### Eigenvector-based distance

The angle between the two eigenvectors was used to compare the direction independently of the magnitude. The distance between the *i*-th eigenvector of one trial *x*_*i*_ and the *j*-th eigenvector of another trial *y*_*j*_ was defined using the cosine distance as $d_{x_{i},y_{j}}^{\mathrm{direction}}=\frac{x_{i}\cdot y_{j}}{∥x_{i}∥∥y_{j}∥}$. Using $d_{x_{i},y_{j}}^{\mathrm{direction}}$, the distance of the controllable direction up to the *m*th eigenmode of a trial pair was determined as follows:

$$D_{x,y}^{\mathrm{direction}}=\frac{\sum_{i=1}^{m}\min(d_{x_{i},y_{1}}^{\mathrm{direction}},d_{x_{i},y_{2}}^{\mathrm{direction}},\ldots ,d_{x_{i},y_{m}}^{\mathrm{direction}})}{m},\;(9)$$

where *m* represents the number of eigenmodes. $D_{x,y}^{\mathrm{direction}}$ is the order-free cosine distance, which uses the distance of the most similar eigenvectors, regardless of the order of the magnitude of the eigenvalues. Since the resulting distance matrix is asymmetric ( $D_{x,y}^{\mathrm{direction}}\neq D_{y,x}^{\mathrm{direction}}$), we symmetrized it by taking the arithmetic mean of each pair of corresponding trials, as follows:

$$D^{\mathrm{direction}}=\frac{D_{x,y}^{\mathrm{direction}}+D_{y,x}^{\mathrm{direction}}}{2}.\;(10)$$

#### Classification of conditions according to inter-trial distance matrices

Each of the three distance matrices we obtained used a different metric, and were thus on different scales and were difficult to compare. Therefore, we performed six-condition classification using the *k*-nearest neighbor method with each distance matrix and obtained a common index of classification performance (*k* = 5). Each trial was classified into one of six conditions, yielding a 6 × 6 confusion matrix. The confusion matrices were averaged across participants. Setting *k* to 1 or 3 and focusing on more local neighborhoods did not change the results.

#### Measure for dimensionality of controllable directions

To verify the dimensionality of the controllability Gramian, the cumulative contribution rate up to the *m*-th eigenmode was determined as follows:

$$\text{Cumulative contribution rate}=\frac{\lambda_{1}+\lambda_{2}+\cdots +\lambda_{i}}{\sum_{i=1}^{m}\lambda_{i}},\;(11)$$

where *λ*_*i*_ is the *i*-th eigenvalue. Rank, which is the number of eigenvectors with eigenvalues above a certain value (e.g., *λ*_·_ > 0.0001), is often used as another measure of dimensionality. When rank is used, factors such as measurement conditions (e.g., number of noisy channels) and preprocessing (e.g., how many independent components were excluded in the preprocessing) that are unrelated to the dimensionality of the controllable space and have no significant impact on the overall signal (eigenvalues are expected to be small) are affected. Therefore, in this study, dimensionality was evaluated using the cumulative contribution ratio, which allowed us to focus only on modes with large eigenvalues.

## Results

Building on the theoretical background of control theory outlined in the Methods section, we first propose a method to estimate the controllability Gramian for evoked neural activity data acquired following an impulse response without explicitly estimating the system’s parameters *A* and *B*. The method itself is well known in standard control theory, but to our knowledge, it has not yet been used in neuroscience applications. Then, we demonstrate the utility of the proposed method by applying it to our TMS-EEG data. By integrating the proposed theoretical framework with empirical data analysis, we investigate the state dependence of the control properties of the EEG dynamics in response to single-shot TMS.

### Proposed method to estimate the controllability Gramian

Here, under the assumption that the external stimulus is an impulse input, we propose a novel method to directly estimate the controllability Gramian, bypassing the need to estimate the system’s parameters. In general, to estimate the controllability Gramian from brain activity data with general external stimulation not restricted to an impulse input, we need to estimate *A* and *B* in Equation 1. Once we have estimated *A* and *B* using some statistical methods, we can compute the controllability Gramian given by Equation 3. However, in the special case where the external input is an impulse input, i.e., the input *u*(*t*) takes a non-zero value only at the time of stimulation (*t* = 0) and then takes zero values for the rest of the time ( [u(0),u(1),…u(m)]=[1,0,…,0]), we do not need to explicitly estimate *A* and *B*, but compute the controllability Gramian directly.

The theoretical validity of this simplification can be demonstrated as follows. If we again introduce $\tilde{x}(t)$ as in the Materials and Methods, and assume that the external stimulus is an impulse input, the time series of neural activity *X* is given by:

$$X=[\tilde{x}(1),\tilde{x}(2),\ldots ,\tilde{x}(m)]=[B,AB,\ldots ,A^{m-1}B].\;(12)$$

Comparing the above equation with the controllability matrix in Equation 4, we can see that the time series of neural activity, *X*, is exactly equal to the controllability matrix C,

$$C=X=[\tilde{x}(1),\tilde{x}(2),\ldots ,\tilde{x}(m)].\;(13)$$

Finally, using Equation 3, we obtain the controllability Gramian as:

$$W=CC^{T},\;(14)$$

$$=XX^{T},\;(15)$$

$$=\left(\begin{array}{ccc} {\tilde{x}}_{1}(1\;:\;m)\cdot {\tilde{x}}_{1}(1\;:\;m) & \ldots & {\tilde{x}}_{1}(1\;:\;m)\cdot {\tilde{x}}_{n}(1\;:\;m)\\ \vdots & \ddots & \vdots \\ {\tilde{x}}_{n}(1\;:\;m)\cdot {\tilde{x}}_{1}(1\;:\;m) & \ldots & {\tilde{x}}_{n}(1\;:\;m)\cdot {\tilde{x}}_{n}(1\;:\;m) \end{array}\right),\;(16)$$

where ${\tilde{x}}_{i}(1\;:\;m)$ is the time series of the *i*-th element from *t* = 1 to *t* = *m*,

$${\tilde{x}}_{i}(1\;:\;m)=[{\tilde{x}}_{i}(1),{\tilde{x}}_{i}(2),\ldots ,{\tilde{x}}_{i}(m)],\;(17)$$

where ${\tilde{x}}_{i}(t)=x_{i}(t)-x_{i}(0)$ (Fig. 1*c*).

### Examples of controllability Gramians from TMS-EEG responses

**Figure 2.**
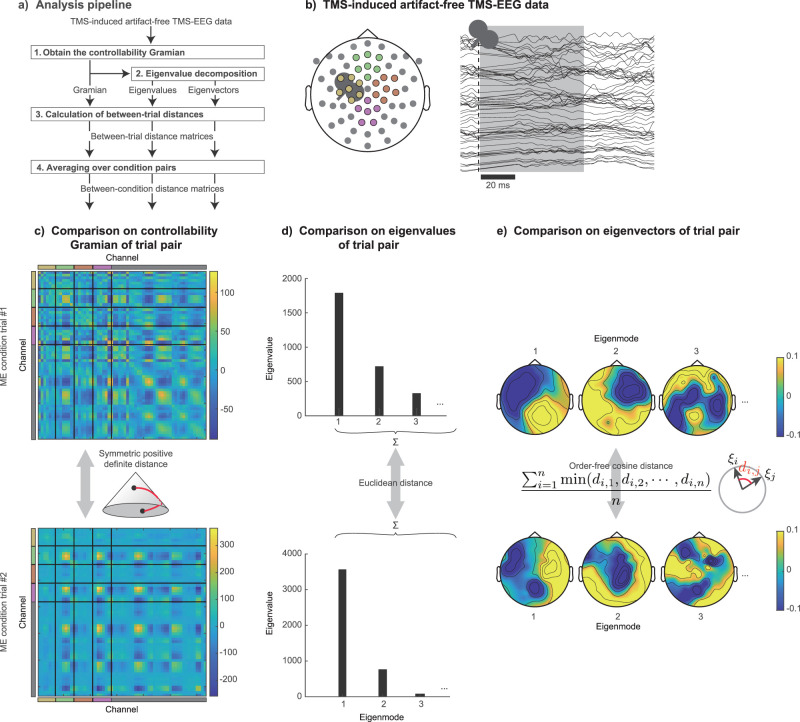
Overview of the proposed methodology and examples single-trial analysis results. ***a***, The controllability Gramian is derived from the single-trial TMS-EEG data after removing TMS-induced artifacts. Subsequently, the eigenvalues and eigenvectors of the controllability Gramian are computed. The comparative analysis entails the comparison of controllability Gramians and summation of their eigenvalues, and eigenvectors across each pair of trials to establish the distance matrices (see ***c*** and Methods). The average distance among trial pairs within the same condition is calculated to generate between-condition distance matrices. ***b***, The left panel displays the electrode arrangement [with colors corresponding to (***c***)] and a sample waveform from a typical single-trial. The temporal segments shaded in gray (1–60 ms from TMS onset) were used for the analysis. Note that the signal from 0 to 20 ms is linearly complemented to remove residual artifacts and the effects of artifact removal. ***c***, The controllability Gramians obtained from the EEG data of two different trials belonging to the same motor execution (ME) condition, are calculated for the distance in symmetric positive definite space. ***d***, The Euclidean distance of the sums was used as the trial-to-trial distance for the eigenvalues. For the eigenvectors **ξ**, the effect of order defined by the magnitude of the eigenvalues was ignored, and only the directions were compared. ***e***, Since eigenvectors are represented by relative values scaled between channels, each trial can be represented as *n* topographies. The distance between the *i*-th eigenvector of trial #1 *ξ*_*i*_ and the *j*-th eigenvector of trial #2 *ξ*_*j*_ was weighed by the cosine distance *d*_*i*,*j*_. The distance between trials was the average of the distances of the closest eigenvector pairs (see Methods). Note that the controllability Gramians, eigenvalues, and eigenvectors illustrated in ***c***–***e*** were obtained by the proposed method applied to two typical trials.

Using the proposed method illustrated in the previous section to compute the controllability Gramian, we here show several examples of controllability Gramian estimated from our TMS-EEG data. The analysis pipeline is summarized in Figure 2*a* (see Methods for details). This study uses a dataset of EEG measurements made during TMS (Table 1). TEPs were recorded with a 63-channel EEG system (*n* = 63, see Materials and Methods). We set the time *t* = 0 to the time of TMS onset, and ***x***(*t*) is a 63-dimensional vector of TEPs at time *t* after the TMS onset. The TMS pulse is considered to be the impulse input, i.e., *u*(0) = 1 and *u*(*t*) = 0 for any *t* ≠ 0. An example of TMS-EEG response data acquired during the ME condition is shown in Figure 2*b*. Then, using the equation for the controllability Gramian under an impulse input (Eq. 16), we obtain an *n* × *n* controllability Gramian matrix from the EEG data after each single-pulse TMS. These matrices are illustrated in Figure 2*c*, where the top and bottom panels show results from two different trials under the ME condition.

### Inter-trial distances based on the controllability Gramian

To explore the extent to which controllability characteristics vary among conditions, we computed the inter-trial distance based on the three metrics: the controllability Gramian-based, eigenvalue-based, and eigenvector-based distances (Fig. 2*c*–*e*; see Methods for details). As explained in the previous section, the controllability Gramian contains all the information for control properties associated with the TMS input. The distance in terms of the controllability Gramian represents the overall difference between trials in terms of control properties. By contrast, the distance for the sum of eigenvalues or eigenvectors represents the difference in terms of partial aspects of control properties, i.e., the overall degree of controllability and the direction in which the system can be controlled.

**Figure 3.**
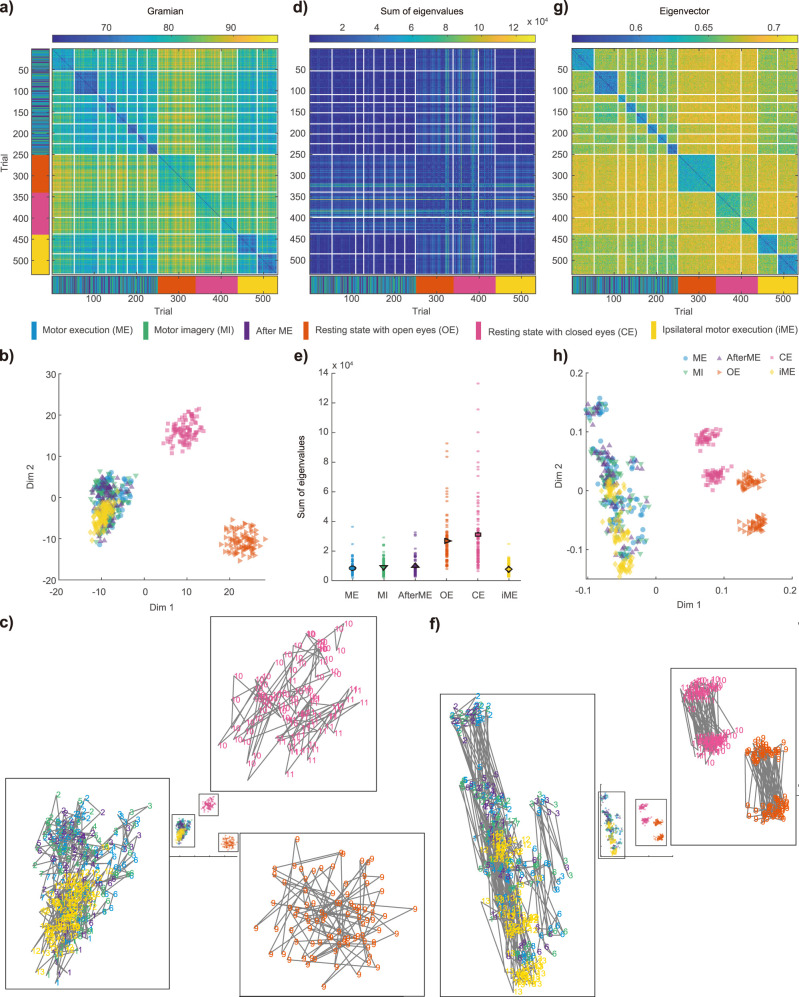
Distances between trials for controllability Gramians, sums of eigenvalues, and eigenvectors for a sample participant. ***a***, A distance matrix of trials for Gramians. Each condition was measured in 100 trials, divided into 1–8 sessions. Note that the number of trials used in the analysis differs between conditions because of the noisy trial removal in the preprocessing. White lines indicate session boundaries and the color bar below indicates the condition to which each trial belongs in (***b***) and (***c***). ***b***, The distance matrix illustrated in (***a***) reduced to a lower dimension using multidimensional scaling. Each point represents one trial, with colors corresponding to different conditions in (***a***). ***c***, To clarify the distances within and between sessions in the same condition, the three clusters in (***b***) are enlarged. Numbers indicate session indices and colors indicate conditions. Consecutive trials are connected by gray lines. ***d***, A distance matrix of trials for sums of eigenvalues. ***e***, The sum of eigenvalues for each trial is represented as a dot, and the trial average is depicted as a colored symbol. ***f***, A distance map of trials for eigenvalues. ***g***, The distance matrix illustrated in (***d***) dimensionally reduced. ***h***, The clusters in (***g***) are enlarged.

Figure 3*a* shows the inter-trial distance matrix based on the controllability Gramian. The indices of the matrix correspond to the trials of the TMS experiments, with each condition corresponding to one of six conditions: ME; MI; AfterME; OE; CE; or iME (Table 1). The trials are ordered from top to bottom according to the order of measurement for each session. The right-hand motor-related task conditions were performed in random order within the same sessions, while the other three conditions were performed in different sessions for each condition (see Materials and Methods for more details). Each session was arranged in the following order: the right-hand motor-related task conditions, i.e., ME, MI, and AfterME, and then OE, CE, and iME. The colors in the matrices represent the distance values, with blue colors representing low difference (i.e., the control properties of the trials are similar) and green to yellowish colors representing high difference, i.e., the control properties of the trials are different. We found that for all conditions, trials within the same sessions were close to each other, as indicated by the relatively small values of the elements of the diagonal blocks corresponding to respective sessions. For a more intuitive understanding of the distances between trials, we have converted the distance matrix shown in Figure 3*a* to scatter plots in low-dimensional space with each trial as a point (Figs. 3*b* and S2). Dimension reduction using multidimensional scaling (MDS) revealed that the two resting conditions (OE and CE) were clearly separated from the other motor-related conditions. In addition, iME was more localized, although it was mixed with the contralateral motor-related condition. It is worth noting that the clusters separated by these conditions span different sessions for each condition (Fig. 3*c*, numbers indicate sessions); that is, beyond intra-session similarity, clusters were identified by similarity between conditions.

In a second step, with the sum of eigenvalues representing the controllable magnitude, we found that a distinct diagonal block structure in the distance matrix was absent, showing that trials within the same session were no closer to each other than trials from different sessions (Figs. 3*d* and S3), unlike situation for the controllability Gramian. While the sum of eigenvalues tended to be higher on average for the two resting conditions, there was considerable trial-to-trial variation (Fig. 3*e*). Thus, compared with the controllability Gramians, the sum of eigenvalues alone less well captures the differences between both conditions and sessions.

In a third step, using the eigenvectors we obtained a distance matrix more-or-less similar to that of the controllability Gramian (which is in contrast to the results for the sum of the eigenvalues), i.e., with the characteristic of small values in the diagonal block components of the distance matrix (Fig. 3*f*). In the reduced-dimensional space achieved by MDS, the ability to distinguish between motor-related and rest conditions in the first dimension aligned with that of the controllability Gramians (Figs. 3*g* and S4). However, in the second dimension there was a wide spread of points representing motor-related conditions (cyan, green, purple, and yellow symbols), with no discernible difference from the resting state conditions (orange and pink symbols). Even within the condition, the trials appeared to be split into multiple subclusters, similar to those found in the controllability Gramian. Both the average distance within a session (ME/MI/AfterME 0.59 ± SD 0.01, OE 0.61 ± 0.02, CE 0.62 ± 0.01, iME 0.59 ± 0.01) and between successive trials (ME/MI/AfterME 0.60 ± 0.01, OE 0.61 ± 0.01, CE 0.63 ± 0.01, iME 0.61 ± 0.01) tended to be longer under the CE condition and a Friedman test revealed a significant effect of condition [within-session *χ*^2^(37) = 3, *p* < 0.001, between successive trials *χ*^2^(28) = 3, *p* < 0.001]. These suggest that one of the reasons for the wider distribution or more clusters of motor-related conditions than the resting state conditions is that data were collected in more sessions (ME/MI/AfterME 4.94 ± 0.90, OE 1.28 ± 0.39, CE 1.18 ± 0.39, iME 1.94 ± 0.56) rather than having large within-session or trial-to-trial variability. These subclusters were to some extent cohesive per session, but were not completely bounded. While they do not mix with each other in the OE, CE, and motor-related conditions, they do mix within the same condition across sessions, indicating a clear difference in controllable direction between conditions (Fig. 3*h*).

The following results were observed. First, there were differences between the resting and motor-related conditions in the Gramians, sum of eigenvalues, and eigenvectors. The sum of eigenvalues was larger in the resting state. Second, Gramians and eigenvectors formed multiple clusters in the lower dimensions. These clusters reflected differences between conditions, which were not solely due to slight variations in the measurements (e.g., electrode impedance and environmental noise), but also due to variations between the conditions. Third, the sum of eigenvectors showed that conditions were closer to each other across sessions. Subclusters were observed within the same condition, which were not observed in the Gramians. Fourth, the three motor-related conditions were more similar to each other than to the resting state conditions in terms of controllability, as indicated by their non-adjacency to the resting state condition trials in the low-dimensional space of Gramians and eigenvectors. While the influence of measurement states could not be completely eliminated, this finding suggests that the motor-related tasks shared common properties between the measurement sessions.

### Inter-condition distances based on the controllability Gramian

**Figure 4.**
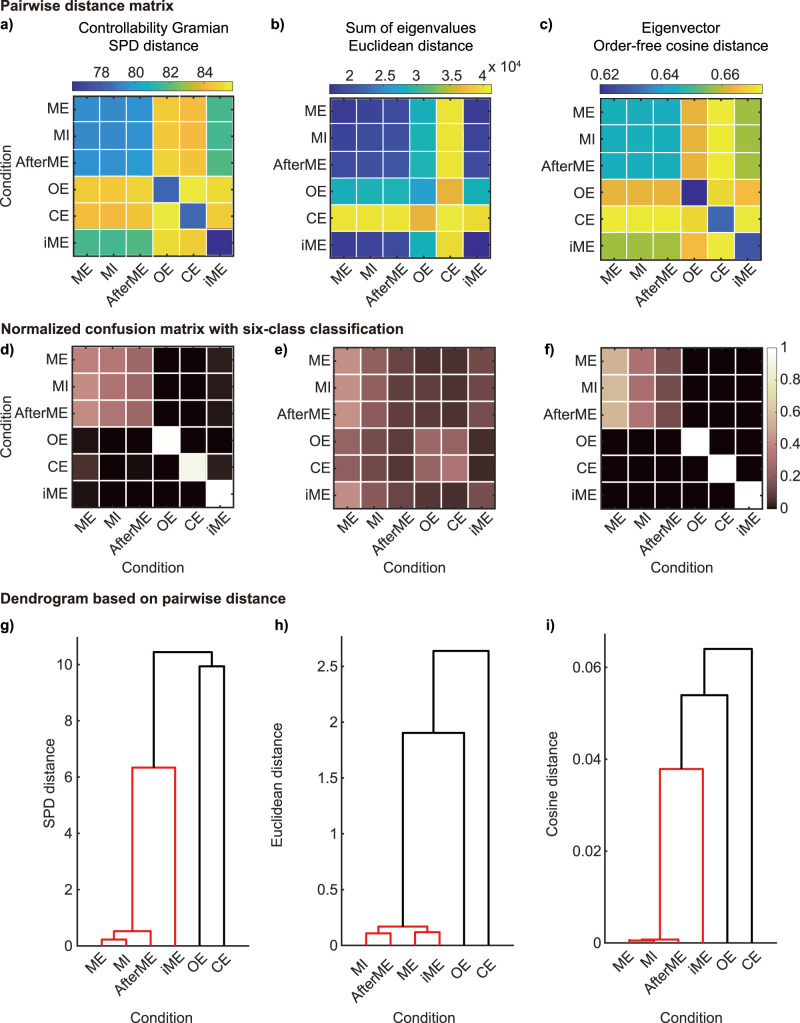
Distance between conditions for the controllability Gramians, sum of their eigenvalues and eigenvectors. ***a***, Distance matrix between-condition pairs for controllability Gramians was averaged over 17 participants. ***b***, Distance matrix between conditions for pairs sums of eigenvalues. ***c***, Distance matrix between conditions for pairs of eigenvectors. ***d***, Normalized confusion matrix for six-class classification using the *k*-nearest neighbor method (*k* = 5) in the distance space defined by (***a***). Rows represent the correct class and the columns the classified class. (***e***) The normalized confusion matrix was obtained from (***b***). ***f***, The normalized confusion matrix was obtained from (***c***). ***g***, Dendrograms of the distance space defined by (***a***). Edges between node pairs that are <70% of the maximum linkage distance are indicated by red lines. ***h***, The dendrogram obtained from (***b***). ***i***, The dendrogram obtained from (***c***).

To observe the average trend of the control properties across participants, we next quantified the averaged discriminability between conditions across participants using the distance matrices of the trials. We first averaged the inter-trial distance matrices for each participant across condition pairs and averaged the condition-based distance matrices across participants, obtaining Figure 4*a*–*c*. The distance matrices are difficult to compare with each other because different distance measures are used for the controllability Gramians, eigenvalues, and eigenvectors. Therefore, we performed six-condition classification using the *k* nearest neighbor method with each distance measure and obtained classification performance as a common measure (Fig. 4*d*–*f*). Additionally, to better illustrate the differences and similarities between conditions, we constructed dendrograms from the distance matrices (Fig. 4*g*–*i*).

First, with the controllability Gramians, we found that the three contralateral motor-related conditions (i.e., ME, MI, and AfterME) were indisputably close to each other in the inter-condition distance matrices (Fig. 4*a*, *d*). The three conditions related to the right-hand motion were the most similar, with the left-hand motion condition being located between the resting conditions and the other motor-related conditions (Fig. 4*g*).

Second, using the eigenvalues, we found that the two resting conditions were distant from the other conditions, as was the case with the controllability Gramians (Fig. 4*b*). However, as seen in Figure 3*e*, the variability between trials made it difficult to classify the conditions in each trial (Fig. 4*e*). The dendrogram based on the distance matrix between conditions showed that iME was most similar to ME, unlike the situation when using the controllability Gramians (Fig. 4*h*).

Third, using the eigenvectors, we found that in addition to the resting condition, which was clearly distinct from the other conditions in the controllability Gramians, iME also showed a trend toward a greater distance from the other conditions (Fig. 4*c*). In terms of classification performance, the three right-hand motor-related conditions were indistinguishable from each other, while the other conditions were not misclassified (as with the controllability Gramians; Fig. 4*f*). The dendrogram showed that iME, like that of the controllability Gramians, was located between the three right-handed motor-related conditions and the two resting conditions, as was the situation for the controllability Gramians (Fig. 4*i*).

Taken together, the results suggest that controllable direction shows greater preservation of the discriminative ability of the controllability Gramians across conditions. Controllable magnitude represents a distinct aspect of variations in brain states to that represented by controllable direction. Ipsilateral and contralateral motion can be distinguished in terms of controllable direction, but not in terms of controllable magnitude. The three distances had one thing in common: four motor-related conditions including iME were closer to each other than 70% of the maximum distance (Fig. 4*g*–*i*, red lines). Additional analyses were conducted and are presented in the supplementary material to address potential concerns regarding trial pairs within the same session and the impact of the time window for analysis (Figs. S5 and S6). These analyses confirm that the observed results are robust, with the concerns identified concerns do not significantly influencing the outcomes.

### Dimensionality of controllable directions

To better understand the internal structure of the state-dependent control properties that we observed, we next investigated the effective dimensionality of the controllable space. This was achieved by examining the cumulative contribution of the eigenvalues of the controllability Gramian, which reveals how the brain’s response to TMS is distributed across different underlying control modes. In short, when a small number of eigenmodes account for a high contribution rate, the dimensionality is considered low, indicating that a few dominant modes can effectively capture the data. In contrast, when a small number of eigenmodes show a low contribution rate, the dimensionality is high, suggesting that the data require a larger number of modes to be accurately represented. As illustrated in the controllability Gramians concept (Fig. 1*b*), high-dimensional controllability Gramians approximate hyperspheres, while low-dimensional ones exhibit a more flattened hyperellipsoidal form. For example, when the dimension is effectively 2 or 3, Gramians correspond to ellipses or ellipsoids. The effective dimensionality of the controllability Gramian is constrained by the underlying system dynamics. Specifically, if the connectivity matrix *A* is low-rank (i.e., the dynamics are dominated by a few modes), the controllable subspace will also be low-dimensional. We demonstrate this direct relationship with a simulation in the Supplementary Text (see “Controllability dimension and the rank of connectivity matrix” and Fig. S7).

**Figure 5.**
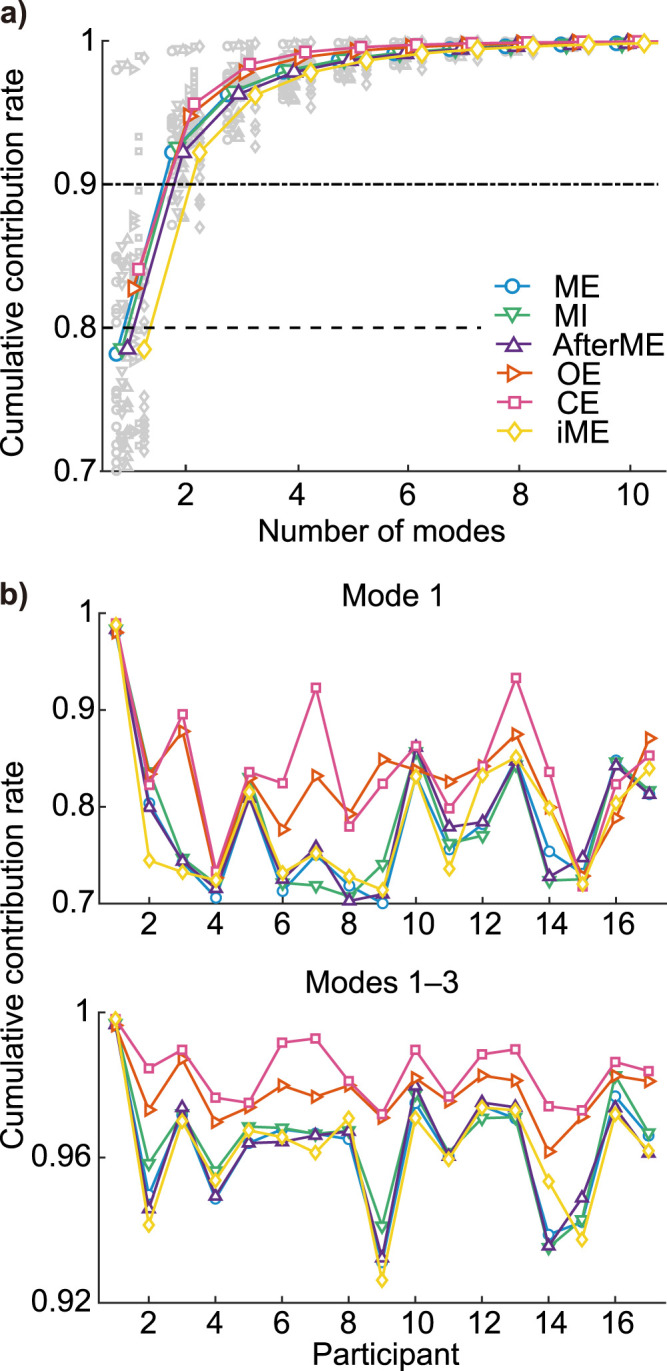
The control properties of M1 under TMS show high cumulative contribution rates for a small number of eigenmodes. ***a***, Colored lines indicate across-participant averages of cumulative contribution rates. The values for each participant are indicated by gray symbols. ***b***, Cumulative contribution rates for the first, and first to third modes. Color and symbols are the same as in ***a***.

We found that in all participants and in all conditions, 90% of the control properties elicited by TMS could be explained by the first to third eigenmode (Fig. 5*a*), indicating the low dimensionality of controllable space. Although there was no common trend among the participants as to which condition gave the highest contribution to the first eigenmode, we found that across a majority of participants, common trends emerged in contribution rates up to eigenmode 3: the contribution rate of CE was closest to 1, while that of OE was closer to 1 than the motor-related conditions, but there were no clear differences between the contribution rates of the motor-related task conditions (Fig. 5*b*). These results suggest that the controllability Gramian can be expressed in about 3 out of 63 dimensions in both resting and motor-related conditions, that resting states are lower-dimensional than motor-related states, and that closing one’s eyes lowers dimensionality even more.

**Figure 6.**
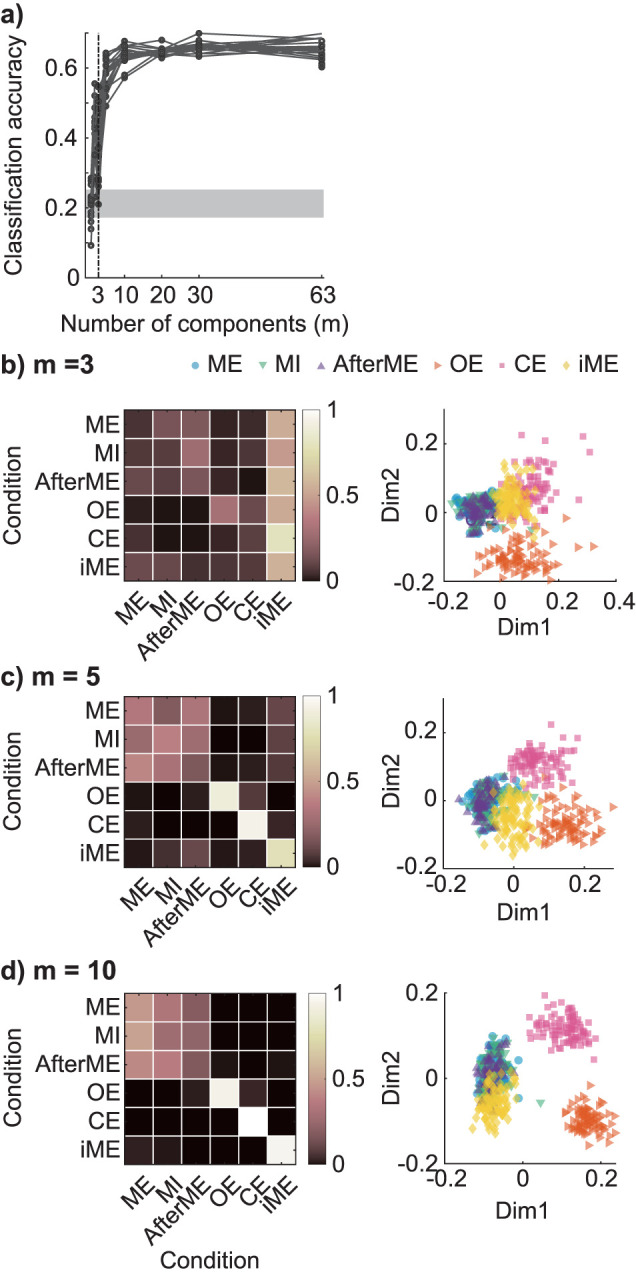
The performance of the six-value classification improved when a fourth or lower mode was added. ***a***, The number of eigenmodes *m* and the percentage that were classified into the correct condition by the *k*-nearest neighbor method (*k* = 5). The black line represents the experimental participants, and gray shading represents the correct response rate obtained when predicting the most sampled condition (i.e., the chance level, the lower line represents the minimum between participants and the upper line represents the maximum). ***b***, Confusion matrices (left) when using the first through to third eigenmodes, which account for 90% of the contribution. Classification performance was calculated for each participant and the participant average is shown. Distribution of trials in the classification space for one typical experimental participant (right). This three-dimensional classification space is represented in two-dimensions through the use of multidimensional scaling. Each point represents a trial. Confusion matrices and typical scatter plots when adding the mode used for condition classification from the first to the fifth (***c***) and first to the tenth (***d***) eigenmodes are shown.

We additionally examined how these few large controllable directions relate to state dependence. Using only the top three eigenvectors, each trial was classified into six conditions, and we found that the correct rate was significantly lower than when all modes were used (Fig. 6). By increasing the number of eigenmodes used to 5, 10, and 30, the correct rate approached that when all modes were used (see Text S6, “Stability analysis of trial-wise controllable directions” and Fig. S8). This suggests that even the lower eigenmodes, which do not provide a high contribution to amplitude restoration, retain information about the differences between conditions.

### Visualizing eigenvectors for interpreting controllable directions

**Figure 7.**
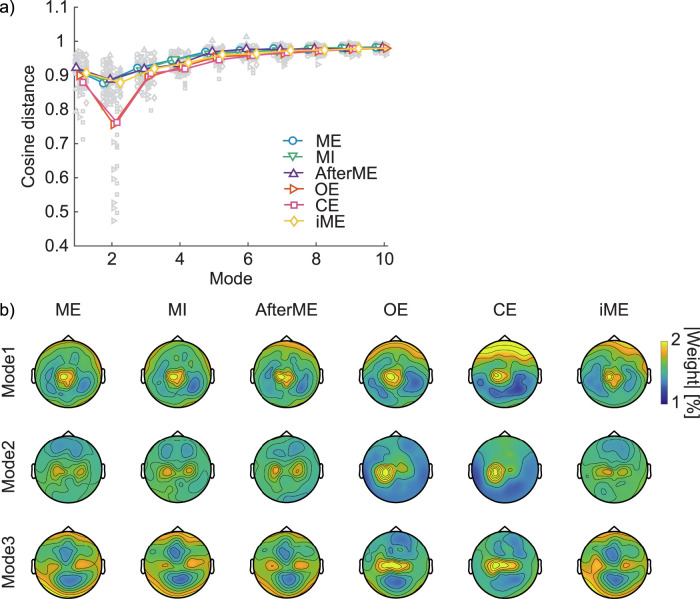
Directional consistency and topographical patterns of controllability Gramian eigenvectors across six conditions. ***a***, Cosine distance between trials indicating the directional consistency of eigenvectors are shown for each of the six experimental conditions. Each small gray symbol represents an individual, while large colored symbols denote the mean across participants for each condition. ***b***, Grand-averaged topographies of the first, second, and third eigenvectors are shown for each condition. At each electrode, the value represents the percentage contribution of the absolute eigenvector weight at that electrode to the total absolute weight across all channels within a trial (summing to 100%), then averaged across trials and participants. Because eigenvector signs are arbitrary, absolute weights were used. For visualization, the color scale is fixed to 1–2% for all panels to facilitate direct comparison across conditions and eigenvectors.

The finding that the three most dominant eigenmodes are insufficient for detailed state classification raises a crucial question: what are the characteristics of these principal directions? To investigate this, we sought to interpret the nature of these controllable directions by visualizing the spatial topographies of their corresponding eigenvectors. Because controllable directions are expected to vary across trials, we first quantified their variability. The mean cosine distance between trials was ≈0.9 for the first eigenvector, reached a minimum at the second eigenvector, and approached 1 as the eigenvector rank increased (Fig. 7*a*). Thus, particularly for higher modes, it is difficult to identify a trial-invariant direction.

**Figure 8.**
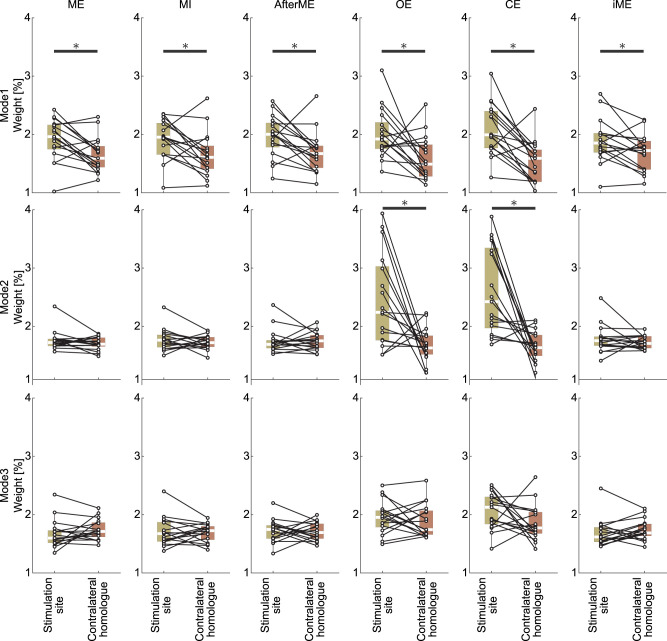
Regional distribution of eigenvector component weights across eigenmodes and experimental conditions. Each boxplot summarizes the percentage contribution of the absolute eigenvector weights at two a priori channel groups: electrodes ipsilateral to the TMS target (C1/CP1/CP3) and their contralateral homologues (C2/CP2/CP4). Percentages are computed relative to the total absolute weight across all channels within each panel. The vertical arrangement of panels corresponds to the rank of the eigenvalue (largest at the top to third largest at the bottom), and the horizontal arrangement corresponds to experimental conditions. In each boxplot, the central white line indicates the median and the box edges indicate the 25th and 75th percentiles; whiskers extend to the most extreme data points not considered outliers (typically within 1.5×IQR). Individual paired observations are overlaid as open circles and connected within participants by lines. Asterisks indicate a significant difference between ipsilateral and contralateral groups within a panel (*p* <0.05, two-tailed Wilcoxon signed-rank test; uncorrected across panels).

2Given this, we focused on the first three eigenvectors—where trial-wise variability is comparatively lower—and visualized grand-averaged topographies of eigenvector weights for each condition (Fig. 7*b*). To emphasize spatial differences, values at each electrode represent the percentage contribution of the absolute weight at that electrode relative to the total across channels within a trial (then averaged across trials and participants). The first eigenvector showed consistently large weights over the stimulation site across conditions, whereas the second and third eigenvectors exhibited reduced stimulation site weighting in some conditions. To test whether lateral bias at the stimulation site characterizes directional differences, we compared weights ipsilateral versus contralateral to TMS using two-tailed Wilcoxon signed-rank tests: the laterality was significant in all conditions for the first eigenvector (all *p* < 0.05 [unc.]), significant only at rest for the second eigenvector (ME *p* = 0.31; MI *p* = 0.46; AfterME *p* = 0.96; OE *p* < 0.05; CE *p* < 0.05; iME *p* = 0.49), and not significant for the third eigenvector (all *p* > 0.05; Fig. 8).

Taken together, these results indicate that the first eigenvector captures a feature common across conditions—prominent weighting at the stimulation site—rather than a condition-specific contrast, consistent with the well-established short-latency TEP peak topographies (P30, P60) typically reported for M1 stimulation. In addition, laterality is maintained at rest for the second eigenvector, which helps explain the rest versus motor-related contrast and is consistent with the differences in contribution rates in Figure 5. By contrast, the condition differences documented earlier—eyes-open versus eyes-closed within rest and right- versus left-hand motor execution (Fig. 4)—were not resolved by the present analysis: neither the grand-averaged eigenvector weight topographies nor the ipsi–contra comparisons yielded separable patterns. These observations suggest that the separability documented in Figures 3 and 4 likely arises from distributed differences in the weight patterns of lower-ranked modes (≥3).

## Discussion

In this study, we propose a simple yet theoretically-grounded method based on control theory for analyzing the control properties of brain dynamics under impulse-like brain stimulation. On the basis of the standard results of the optimal control problem, we can regard the time series of neural activity as a controllability matrix. This enables us to simply compute the controllability Gramian without directly inferring the model parameters of linear dynamical systems. This simplification relies on several key assumptions (discussed in detail in Text S7; Slotine and Li, 1991; Tseng et al., 1995; Sontag, 1998; Khalil, 2001; Isidori, 2006; Chang et al., 2012; Jiang and Lai, 2019; Shakeel et al., 2021), which enables us to simply compute the controllability Gramian without directly inferring the model parameters of linear dynamical systems. We applied our method to single-shot single-trial TMS-EEG data and used the controllability Gramian and its eigenvalues and eigenvectors to examine the differences and similarities in control properties across conditions. These control properties give a reasonable interpretation of responsiveness to local stimuli: the controllability Gramian represents the controllable space with unit input, its eigenvectors represent the controllable direction, and the corresponding eigenvalues represent the controllable magnitude. We found that controllability Gramians estimated from measured signals contains information that allowed distinguishing between open-eye rest, closed-eye rest, contralateral motor-related states, and ipsilateral motor execution state. This distinction is brought about by the controllable direction rather than the controllable magnitude. The cumulative contribution of eigenvalues indicated that the resting state had less response complexity, regardless of eye opening or eye closing. These results suggest that the state dependence of the EEG response immediately after TMS is characterized by spread rather than amplitude.

### Novel perspectives of the proposed control theory-based measure on perturbation approaches

Although the proposed control theory-based measure is related to some measures quantified in previous studies, its main novel contribution lies in its ability to unify two aspects: the directions in which brain dynamics can be controlled and the extent to which they can be controlled with a fixed amount of input. In terms of relationships with previous studies, excitability and connectivity (Siebner et al., 2009; Bergmann et al., 2016) have previously been quantified as the magnitude of the response activity to a stimulus and the extent to which stimulus-evoked activity propagates (Casali et al., 2010, 2013; Casarotto et al., 2016; Comolatti et al., 2019). The excitability and connectivity quantified in these previous studies are related to the sum of eigenvalues of the controllability Gramian in our framework, which quantifies the overall degree of propagation of the stimulus-evoked response. However, the eigenvector we focused on indicates the specific direction of this propagation. In previous explorations of control properties from measurement signals, only the eigenvalues were utilized and the corresponding eigenvectors were neglected (Gu et al., 2015), or they were used as eigenmodes without being separated (Comolatti et al., 2019). In this study, we showed that differences between conditions clearly appear in controllable directions rather than in controllable magnitudes, indicating the importance of investigating both aspects.

### Interpretability of controllable directions in EEG data

Similarly, to interpret controllability measures in light of known EEG phenomena, it is important to consider how they relate to established markers such as event-related potentials (ERPs) and induced oscillations. The controllability magnitude may partially reflect the ERP amplitude, as both indicate the strength of neural responses to external perturbations. However, latency-specific features, such as the timing of ERP components, are not explicitly captured by the Gramian-based framework. This is because the controllability analysis reflects the temporal evolution of the signal across a predefined time window and does not isolate particular moments in time. Similarly, frequency-specific information is not directly accessible in the current formulation, since the controllability metric integrates signal characteristics across all frequency bands without decomposing them. As a future direction, incorporating signal decomposition techniques such as dynamic mode decomposition, which enables the reconstruction of frequency-specific components, could allow the controllability analysis to align more directly with known neural dynamics. This would open new avenues for interpreting how control properties relate to oscillatory activity at different frequency ranges, potentially enhancing the neurophysiological validity of this approach.

A further limitation lies in the interpretability of controllable directions in terms of known neuroanatomical and functional networks. While our eigenvector-based analysis revealed condition-specific spatial patterns at the sensor level, the current approach does not directly map these directions onto cortical regions or large-scale brain networks, such as the motor system or default mode network. This is in part due to our use of EEG data in sensor space, without source localization or cortical parcellation. As a result, although we observed consistent spatial motifs (e.g., stronger controllability weights near the stimulation site), we are unable to directly compare these patterns to established functional circuits or to assess their neurobiological relevance in a network-theoretic context. Bridging this gap will require future work incorporating source-level estimation and anatomically informed priors, which could allow controllable directions to be interpreted in terms of canonical brain systems and cognitive functions. Such developments will be essential for leveraging the theoretical framework of network control to make deeper neuroscientific inferences.

### Neurophysiological interpretation of results in relation to previous studies

We found that the controllable magnitude fluctuates more during rest conditions than during motor-related conditions. One possible explanation is the reduced variability in brain activity induced by the visual stimuli used for task instructions, which stabilizes neural activity by constraining it to a specific trajectory (He, 2011; He and Zempel, 2013). In addition, both motor execution and motor imagery enhance inter-trial coherence within the consistent activity of task-positive networks such as the somatomotor network (Churchland et al., 2010). By contrast, during resting states, neural networks are less constrained by external demands, allowing greater flexibility and spontaneous variation between interconnected networks. This network-level flexibility may account for the higher trial-to-trial variability observed in resting state EEG.

Our analysis further reveals a clear dissociation between controllable directions and controllable magnitudes in their ability to identify brain states. This contrast is conceptually coherent with the respective roles of eigenvectors and eigenvalues in control theory: while eigenvectors characterize the directions in state space where control is exerted, eigenvalues reflect the magnitude of control energy required along those directions. The controllable direction serves as a relevant indicator of spatial topography because it captures the relative relationships between channels. Although individual eigenvectors vary across trials, treating them as a collective subspace allows for stable identification of brain state–specific directional patterns (Text S8). The present findings suggest that the directional structure of controllability, rather than its overall magnitude, may carry more informative signatures of brain state in a stimulation-based EEG setting. This aligns with recent theoretical proposals that emphasize the importance of considering both the geometry and efficiency of control (Gu et al., 2015; Deng et al., 2022).

Analysis based on controllable directions revealed that the four motor-related conditions were clearly separable from the two resting state conditions. The latter were characterized by eigenvector weights concentrated around the stimulation site, which contributed to their distinction from motor-related states. The two resting conditions could also be differentiated from each other, likely reflecting known differences in visual evoked potentials and EEG topographies between open-eye and closed-eye states (Allison et al., 1977; Barry et al., 2007). Among the motor-related conditions, right- and left-hand movements were distinguishable, consistent with previous brain–computer interface studies exploiting topographic differences between effectors (Huang et al., 2009; Xu et al., 2011). This distinction is not attributable to a simple lateral reversal of topography, as our method does not consider the sign of the eigenvector, suggesting that the two movements engage different neural activation patterns.

By contrast, motor execution and motor imagery conditions could not be separated, consistent with the fact that motor imagery can modulate M1 excitability in ways similar to motor execution (Takemi et al., 2013). Previous EEG studies have reported reliable differences between execution and imagery in terms of sensorimotor desynchronization and spatial topographies (Neuper et al., 2005; Zabielska-Mendyk et al., 2018), indicating that the lack of separation here should not be taken as evidence of neurophysiological equivalence. Instead, it may reflect limitations of the current perturbation-based framework, which captures brain responses to brief, localized input but may not detect subtler differences in endogenous network activity. Such distinctions might emerge more clearly under alternative experimental configurations, such as varying stimulation sites, input characteristics (e.g., intensity, duration, waveform; Ogino et al., 2025), or incorporating task-informed priors into the control model. Combining this approach with source-level EEG analysis or complementary modalities (e.g., EMG and fMRI) could also help resolve finer-grained differences between execution- and imagery-related brain states.

A possible explanation for the lower-dimensional controllability observed in resting state conditions is stronger and more globally synchronized neural activity. Biswal et al. (1995) showed that motor-related regions exhibit significant functional connectivity even at rest, suggesting the presence of intrinsic large-scale synchrony. While task engagement can reconfigure and strengthen specific synchrony patterns (Manganotti et al., 1998; Jiang et al., 2004), the more uniform and stereotyped coupling seen at rest may reduce the diversity of response modes. This could lead to fewer distinct controllable directions, resulting in lower-dimensional controllability despite high overall controllability magnitude.

Taken together, our approach reveals that some brain states, such as motor-related task versus resting states, can be well distinguished by the properties of the controllability Gramian, whereas other states, such as motor execution and motor imagery, cannot be so differentiated. Future work is needed to verify which factors of brain states are reflected in the controllability associated with this TMS-EEG framework and which factors are not, with this involving performing TMS-EEG under more diverse conditions.

## References

[B1] Allison T, Matsumiya Y, Goff GD, Goff WR (1977) The scalp topography of human visual evoked potentials. Electroencephalogr Clin Neurophysiol 42:185–197. 10.1016/0013-4694(77)90025-6 65254

[B2] Awiszus F (2003) TMS and threshold hunting. Suppl Clin Neurophysiol 56:13–23. 10.1016/0013-4694(77)90025-6 14677378

[B3] Barry RJ, Clarke AR, Johnstone SJ, Magee CA, Rushby JA (2007) EEG differences between eyes-closed and eyes-open resting conditions. Clin Neurophysiol 118:2765–2773. 10.1016/j.clinph.2007.07.028 17911042

[B4] Beck MM, Heyl M, Mejer L, Vinding MC, Christiansen L, Tomasevic L, Siebner HR (2024) Methodological choices matter: a systematic comparison of TMS-EEG studies targeting the primary motor cortex. Hum Brain Mapp 45:e70048. 10.1002/hbm.70048 39460649 PMC11512442

[B5] Bergmann TO, Karabanov A, Hartwigsen G, Thielscher A, Siebner HR (2016) Combining non-invasive transcranial brain stimulation with neuroimaging and electrophysiology: current approaches and future perspectives. Neuroimage 140:4–19. 10.1016/j.neuroimage.2016.02.012 26883069

[B6] Bhatia R (2009) Positive definite matrices. Princeton: Princeton University Press.

[B7] Biabani M, Fornito A, Mutanen TP, Morrow J, Rogasch NC (2019) Characterizing and minimizing the contribution of sensory inputs to TMS-evoked potentials. Brain Stimul 12:1537–1552. 10.1016/j.brs.2019.07.009 31377097

[B8] Biswal B, Yetkin FZ, Haughton VM, Hyde JS (1995) Functional connectivity in the motor cortex of resting human brain using echo-planar MRI. Magn Reson Med 34:537–541. 10.1002/mrm.1910340409 8524021

[B9] Brunton SL, Kutz JN (2019) Data-driven science and engineering: machine learning, dynamical systems, and control. Cambridge: Cambridge University Press.

[B10] Casali AG, Casarotto S, Rosanova M, Mariotti M, Massimini M (2010) General indices to characterize the electrical response of the cerebral cortex to TMS. Neuroimage 49:1459–1468. 10.1016/j.neuroimage.2009.09.026 19770048

[B11] Casali AG, et al. (2013) A theoretically based index of consciousness independent of sensory processing and behavior. Sci Transl Med 5:198ra105. 10.1126/scitranslmed.3006294 23946194

[B12] Casarotto S, et al. (2016) Stratification of unresponsive patients by an independently validated index of brain complexity. Ann Neurol 80:718–729. 10.1002/ana.24779 27717082 PMC5132045

[B13] Chang J-Y, Pigorini A, Massimini M, Tononi G, Nobili L, Van Veen BD (2012) Multivariate autoregressive models with exogenous inputs for intracerebral responses to direct electrical stimulation of the human brain. Front Hum Neurosci 6:317. 10.3389/fnhum.2012.00317 23226122 PMC3510687

[B14] Churchland MM, et al. (2010) Stimulus onset quenches neural variability: a widespread cortical phenomenon. Nat Neurosci 13:369–378. 10.1038/nn.2501 20173745 PMC2828350

[B15] Comolatti R, et al. (2019) A fast and general method to empirically estimate the complexity of brain responses to transcranial and intracranial stimulations. Brain Stimul 12:1280–1289. 10.1016/j.brs.2019.05.013 31133480

[B16] Conde V, Tomasevic L, Akopian I, Stanek K, Saturnino GB, Thielscher A, Bergmann TO, Siebner HR (2019) The non-transcranial TMS-evoked potential is an inherent source of ambiguity in TMS-EEG studies. Neuroimage 185:300–312. 10.1016/j.neuroimage.2018.10.052 30347282

[B17] Deng S, Li J, Thomas Yeo BT, Gu S (2022) Control theory illustrates the energy efficiency in the dynamic reconfiguration of functional connectivity. Commun Biol 5:295. 10.1038/s42003-022-03196-0 35365757 PMC8975837

[B18] Gu S, et al. (2015) Controllability of structural brain networks. Nat Commun 6:8414. 10.1038/ncomms9414 26423222 PMC4600713

[B19] Gu S, Betzel RF, Mattar MG, Cieslak M, Delio PR, Grafton ST, Pasqualetti F, Bassett DS (2017) Optimal trajectories of brain state transitions. Neuroimage 148:305–317. 10.1016/j.neuroimage.2017.01.003 28088484 PMC5489344

[B20] He BJ (2011) Scale-free properties of the functional magnetic resonance imaging signal during rest and task. J Neurosci 31:13786–13795. 10.1523/JNEUROSCI.2111-11.2011 21957241 PMC3197021

[B21] He BJ, Zempel JM (2013) Average is optimal: an inverted-U relationship between trial-to-trial brain activity and behavioral performance. PLoS Comput Biol 9:e1003348. 10.1371/journal.pcbi.1003348 24244146 PMC3820514

[B22] Herring JD, Thut G, Jensen O, Bergmann TO (2015) Attention modulates TMS-locked alpha oscillations in the visual cortex. J Neurosci 35:14435–14447. 10.1523/JNEUROSCI.1833-15.2015 26511236 PMC4623224

[B23] Huang D, Lin P, Fei D-Y, Chen X, Bai O (2009) Decoding human motor activity from EEG single trials for a discrete two-dimensional cursor control. J Neural Eng 6:046005. 10.1088/1741-2560/6/4/046005 19556679

[B24] Isidori A (2006) Nonlinear control systems, Ed 3. Communications and control engineering series. New York, NY: Springer.

[B25] Jakowluk W (2024) Optimal input signal design in control systems identification. Bialystok: Bialystok University of Technology.

[B26] Jiang J, Lai Y-C (2019) Irrelevance of linear controllability to nonlinear dynamical networks. Nat Commun 10:3961. 10.1038/s41467-019-11822-5 31481693 PMC6722065

[B27] Jiang T, He Y, Zang Y, Weng X (2004) Modulation of functional connectivity during the resting state and the motor task. Hum Brain Mapp 22:63–71. 10.1002/hbm.20012 15083527 PMC6871844

[B28] Kamiya S, Kawakita G, Sasai S, Kitazono J, Oizumi M (2023) Optimal control costs of brain state transitions in linear stochastic systems. J Neurosci 43:270–281. 10.1523/JNEUROSCI.1053-22.2022 36384681 PMC9838695

[B29] Kawakita G, Kamiya S, Sasai S, Kitazono J, Oizumi M (2022) Quantifying brain state transition cost via Schrödinger bridge. Netw Neurosci 6:118–134. 10.1162/netn_a_00213 35356194 PMC8959122

[B30] Khalil HK (2001) Nonlinear systems, Ed 3. Upper Saddle River, NJ: Pearson.

[B31] Lee M, et al. (2022) Quantifying arousal and awareness in altered states of consciousness using interpretable deep learning. Nat Commun 13:1064. 10.1038/s41467-022-28451-0 35217645 PMC8881479

[B32] Manganotti P, Gerloff C, Toro C, Katsuta H, Sadato N, Zhuang P, Leocani L, Hallett M (1998) Task-related coherence and task-related spectral power changes during sequential finger movements. Electroencephalogr Clin Neurophysiol 109:50–62. 10.1016/S0924-980X(97)00074-X 11003064

[B33] Medaglia JD, Pasqualetti F, Hamilton RH, Thompson-Schill SL, Bassett DS (2017) Brain and cognitive reserve: translation via network control theory. Neurosci Biobehav Rev 75:53–64. 10.1016/j.neubiorev.2017.01.016 28104411 PMC5359115

[B34] Neuper C, Scherer R, Reiner M, Pfurtscheller G (2005) Imagery of motor actions: differential effects of kinesthetic and visual-motor mode of imagery in single-trial EEG. Brain Res Cogn Brain Res 25:668–677. 10.1016/j.cogbrainres.2005.08.014 16236487

[B35] Ogino M, Sekizawa D, Kitazono J, Oizumi M (2025) Designing optimal perturbation inputs for system identification in neuroscience. bioRxiv, 2025.03.02.641008.

[B36] Okazaki YO, Mizuno Y, Kitajo K (2020) Probing dynamical cortical gating of attention with concurrent TMS-EEG. Sci Rep 10:1–10. 10.1038/s41598-020-61590-2 32188883 PMC7080792

[B37] Oldfield RC (1971) The assessment and analysis of handedness: the Edinburgh inventory. Neuropsychologia 9:97–113. 10.1016/0028-3932(71)90067-4 5146491

[B38] Oostenveld R, Fries P, Maris E, Schoffelen J-M (2011) FieldTrip: open source software for advanced analysis of MEG, EEG, and invasive electrophysiological data. Comput Intell Neurosci 2011:156869. 10.1155/2011/156869 21253357 PMC3021840

[B39] Rocchi L, Di Santo A, Brown K, Ibáñez J, Casula E, Rawji V, Di Lazzaro V, Koch G, Rothwell J (2021) Disentangling EEG responses to TMS due to cortical and peripheral activations. Brain Stimul 14:4–18. 10.1016/j.brs.2020.10.011 33127580

[B40] Rogasch NC, Sullivan C, Thomson RH, Rose NS, Bailey NW, Fitzgerald PB, Farzan F, Hernandez-Pavon JC (2017) Analysing concurrent transcranial magnetic stimulation and electroencephalographic data: a review and introduction to the open-source TESA software. Neuroimage 147:934–951. 10.1016/j.neuroimage.2016.10.031 27771347

[B41] Rose NS, LaRocque JJ, Riggall AC, Gosseries O, Starrett MJ, Meyering EE, Postle BR (2016) Reactivation of latent working memories with transcranial magnetic stimulation. Science 354:1136–1139. 10.1126/science.aah7011 27934762 PMC5221753

[B42] Shakeel A, Onojima T, Tanaka T, Kitajo K (2021) Real-time implementation of EEG oscillatory phase-informed visual stimulation using a least mean square-based AR model. J Pers Med 11:38. 10.3390/jpm11010038 33440652 PMC7828009

[B43] Shikauchi Y, Uehara K, Okazaki YO, Kitajo K (2025) Electroencephalographic responses before, during, and after upper limb paired associative stimulation. Data Brief 60:111467. 10.1016/j.dib.2025.111467 40226202 PMC11986603

[B44] Siebner HR, et al. (2009) Consensus paper: combining transcranial stimulation with neuroimaging. Brain Stimul 2:58–80. 10.1016/j.brs.2008.11.002 20633405

[B45] Slotine J-JE, Li W (1991) Applied nonlinear control. Englewood Gliffs: Prentice-Hall.

[B46] Sontag ED (1998) Mathematical control theory: deterministic finite dimensional systems, Ed 2. Texts in applied mathematics. New York, NY: Springer.

[B47] Takemi M, Masakado Y, Liu M, Ushiba J (2013) Event-related desynchronization reflects downregulation of intracortical inhibition in human primary motor cortex. J Neurophysiol 110:1158–1166. 10.1152/jn.01092.2012 23761697

[B48] Tseng SY, Chen RC, Chong FC, Kuo TS (1995) Evaluation of parametric methods in EEG signal analysis. Med Eng Phys 17:71–78. 10.1016/1350-4533(95)90380-T 7704347

[B49] Xu P, Yang P, Lei X, Yao D (2011) An enhanced probabilistic LDA for multi-class brain computer interface. PLoS One 6:e14634. 10.1371/journal.pone.0014634 21297944 PMC3031502

[B50] You K, Park H-J (2021) Re-visiting Riemannian geometry of symmetric positive definite matrices for the analysis of functional connectivity. Neuroimage 225:117464. 10.1016/j.neuroimage.2020.117464 33075555

[B51] Zabielska-Mendyk E, Francuz P, Jaśkiewicz M, Augustynowicz P (2018) The effects of motor expertise on sensorimotor rhythm desynchronization during execution and imagery of sequential movements. Neuroscience 384:101–110. 10.1016/j.neuroscience.2018.05.028 29852241

